# Perception of a need to change weight in individuals living with and beyond breast, prostate and colorectal cancer: a cross-sectional survey

**DOI:** 10.1007/s11764-023-01333-0

**Published:** 2023-01-26

**Authors:** Gabriella N. Heuchan, Phillippa J. Lally, Rebecca J. Beeken, Abigail Fisher, Rana E. Conway

**Affiliations:** 1 Research Department of Behavioural Science and Health, University College London, Gower Street, London, WC1E 6BT UK; 2 Leeds Institute of Health Sciences, University of Leeds, Leeds, LS2 9JT UK; 3 Department of Psychology, University of Surrey, Guildford, Surrey GU2 7HX UK

**Keywords:** Cancer, Cancer survivor, BMI, Obesity, Weight loss

## Abstract

**Purpose:**

People living with and beyond cancer (LWBC) are advised to achieve a body mass index (BMI) within the healthy range (≥ 18.5 and < 25). Not perceiving a need for weight change may be a barrier to achieving a healthy weight. This study aimed to explore factors associated with perceived need for weight change among people LWBC.

**Methods:**

Adults diagnosed with breast, prostate, or colorectal cancer were recruited through National Health Service sites in Essex and London. Participants (*N* = 5835) completed the ‘Health and Lifestyle After Cancer’ survey, which included a question on perceived need to change weight. Associations between perceived need for weight change and BMI, and perceived need for weight change and health and demographic variables, were analyzed using chi-square tests and logistic regression, respectively.

**Results:**

The proportion of participants perceiving a need to lose weight differed according to BMI category: healthy weight (23%), overweight (64%), obese (85%) (*P* < 0.001). Having overweight or obesity but not perceiving a need to lose weight was associated with being older, male, non-white, not married or cohabiting, and having cancer that had spread, no formal qualifications, no comorbidities, and having received chemotherapy.

**Conclusions:**

Perceived need to lose weight is prevalent among people LWBC with obesity and overweight. This group may be interested in weight management support. Demographic and health factors were associated with having obesity or overweight but not perceiving a need to lose weight.

**Implications for cancer survivors:**

Weight loss interventions for people LWBC are needed. A subset of people LWBC with overweight and obesity may need additional information or motivators to engage with weight management.

**Supplementary Information:**

The online version contains supplementary material available at 10.1007/s11764-023-01333-0.

## Introduction

The number of people living with and beyond cancer (LWBC) is increasing worldwide due to improvements in cancer care and diagnosis. In the United Kingdom, the estimated number of people LWBC is expected to exceed 4 million by 2030 [1]. However, quality of life and longevity of individuals after a cancer diagnosis can be affected by comorbidities, including overweight and obesity.

There is evidence that overweight and obesity at cancer diagnosis [2–4], as well as weight gain post-diagnosis [3, 5, 6] are associated with increased risk of all-cause mortality and cancer recurrence for most cancer types, including breast and colorectal cancer [4]. A recent systematic review and meta-analysis found that individuals with obesity had significantly worse odds of overall survival (HR, 1.14; 95% CI, 1.09–1.19), cancer-specific survival (HR, 1.17; 95% CI, 1.12–1.23), and disease or progression-free survival (HR, 1.13; 95% CI, 1.07–1.19) than individuals without obesity [4]. This may be partly due to effects of cancer therapies. For example, some radiation and chemotherapies are associated with increased risk of cardiovascular disease (CVD) [7, 8], and this risk is heightened in individuals with obesity and its associated conditions, including hypertension and diabetes [8]. As cancer survivors have considerably increased risk of CVD mortality compared to the general population [9], strategies to address modifiable cancer and CVD risk factors such as excess weight in this population are vital.

The World Cancer Research Fund (WCRF) lists keeping [your] weight within the healthy body mass index (BMI) range of 18.5–24.9 kg/m^2^ among its recommendations for primary and secondary cancer prevention [10], and public health messaging and campaigns have highlighted excess weight as a modifiable risk factor for cancer [11], and other non-communicable diseases (NCDs) [12]. However, these interventions have not led to reductions in either the prevalence of excess weight nor NCDs associated with weight and lifestyle [13, 14]. A number of factors may contribute to this, for example, poor intervention design and delivery. There has been criticism of some public health campaigns for failing to employ evidence-based techniques based on behavior change theory, including goal setting, self-monitoring, and building self-efficacy [15], as well as failing to tackle the breadth of risk factors which can contribute to obesity, including health inequalities and genetics [16, 17]. Nevertheless, there is evidence that tailored weight loss interventions for people LWBC can be beneficial for participants, with a recent Cochrane review concluding that women living with and beyond breast cancer with a BMI ≥ 25 participating in weight loss interventions were more likely to lose weight, reduce their waist circumference and BMI, and improve well-being [18].

An important determinant of engagement in potentially effective interventions, though not necessarily achieving sustained weight loss, is recognizing a need to manage one’s own weight. It is possible that individuals with overweight or obesity who do not perceive themselves as being in at-risk categories regarding their bodyweight do not perceive a need for weight loss, and do not take action to change their weight. This is supported by evidence that self-perception of adiposity, but not BMI, predicts attempts to lose weight [19]. Previous studies have found that both men [20] and women [21] tend to misperceive their weight status, with underestimation of weight status appearing to be associated with increasing age [21] and weight [21, 22], lower level of education [21–23], and being male [22, 24, 25]. However, there are limited studies assessing individuals’ perceptions of weight in patient populations. For example, people LWBC may be less likely to perceive a need to lose weight due to an emphasis on prevention of unintentional weight loss and nutritional interventions to maintain or increase weight in cancer care pathways [26].

This study aimed to investigate whether individuals in different BMI categories perceive a need to change their weight, and to identify factors associated with these perceptions among those living with and beyond breast, prostate, and colorectal cancer. The study also aimed to identify demographic and health factors associated with not perceiving a need to lose weight in people LWBC with overweight and obesity.

## Methods

### Design

This cross-sectional study used data from the ‘Health and Lifestyle After Cancer’ survey [27]. The questionnaire included questions about demographics, health, physical activity, diet and nutrition, alcohol, and sleep. Further details of the survey can be found in the protocol paper for the Advancing Survival Cancer Outcomes Trial (ASCOT) [27] as this survey was used to identify potential trial participants.

### Participants

Participants were recruited through 10 participating NHS Trusts across London and Essex, through the Clinical Research Network Portfolio. Participating sites posted letters of invitation, the survey in paper form, and a link to an internet version of the survey to the eligible patients. Hospitals were asked to invite patients aged 18 years and older who had received a diagnosis of breast, prostate or colorectal cancer between 2012 and 2015. These dates were chosen to make the numbers manageable and in the hope of reaching people who had completed, or were close to completing, primary curative treatment (an inclusion criteria for the ASCOT trial), though participants who had been diagnosed outside of these dates and returned surveys were still included in the current analysis. Participants in the final sample, were diagnosed with breast, prostate or colorectal cancer between 1994 and 2017 (mean time since cancer diagnosis, 36.1 months, SD = 13.89). However, as some participants were diagnosed with a subsequent cancer, the range of diagnosis dates for participant’s most recent cancer was 2000–2017 (mean time since most recent cancer diagnosis 35.5 months, SD = 13.6). Survey inclusion criteria were deliberately broad to reduce burden on hospital sites and because we were interested in the views of anyone living with and beyond breast, colorectal, and prostate cancer.

Participants completed the ‘Health and Lifestyle after Cancer Survey’ on paper or online and returned it directly to the research team. Returned questionnaires were accepted between February 2015 and January 2018.

Ethical approval was obtained through the National Research Ethics Service Committee South Central—Oxford B (reference number 14/SC/1369).

### Measures

#### Dependent variable

Participants’ perception of a need to change their weight was assessed by the question ‘Which of the following best describes you at the present time? (a) I think I should be trying to lose weight; (b) I think I should be trying to gain weight; (c) I don’t think I need to change my weight; (d) Don’t know’.

#### Independent variables

##### BMI

Participants self-reported their height and weight as part of the survey. From these data BMI (kg/m^2^) was calculated, and participants were categorized as underweight (BMI < 18.5), healthy weight (BMI ≥ 18.5 and < 25), overweight (BMI ≥ 25 and < 30), and obese (BMI ≥ 30) [28].

##### Demographics

Participants were asked their age in years, and their highest level of education achieved. A range of responses were presented to capture the full range of qualifications people can have and the responses were then coded into (a) none, (b) GCSE/vocational, (c) A-Level, (d) degree or higher. The survey also collected data on marital status and responses were dichotomised into married/co-habiting or divorced/separated/widowed/single after multiple imputation. Data collected on participants’ ethnicity was also dichotomised into white or non-white, as only 554 (9.5%) participants selected a non-white ethnicity.

##### Health

Participants were asked whether their cancer had spread to other parts of their body. Possible responses were (a) yes, (b) no, or (c) don’t know. Before multiple imputation, ‘don’t know’ was recoded as missing. To gather data on comorbidities, the questionnaire also included a list of 15 conditions for participants to select from, and provided a free text box to add any other conditions. Responses were reviewed and total number of comorbidities for each participant was calculated. Participants were also asked to select what treatments they had received for their cancer.

### Statistical analysis

Statistical analyses were performed in IBM SPSS version 28. Descriptive statistics were calculated for demographic and health variables and BMI.

Chi-square tests were performed to compare proportions of participants in each BMI category by response to the survey question on perceived need to change weight. Among participants with a BMI ≥ 25, logistic regression analysis was used to explore factors associated with not perceiving a need to lose weight. For the regression analysis, responses to the question on perceived need to lose weight were dichotomised into perceiving a need to lose weight (selected option ‘a’) or not perceiving a need to lose weight (selected option b, c, or d). The independent variables used in the logistic regression analysis were age in years (continuous), sex (male/female), ethnicity (white/non-white), marital status (married/cohabiting or single/widow), highest level of education (4 levels), cancer spread (yes/no), number of comorbidities (0/1/2/3 +), and four variables for treatment type (surgery/chemotherapy/radiotherapy/hormonal therapy).

Chi-square and logistic regression analysis were run on multiply imputed data with five imputations. Predictor variables for the multiple imputation, in order of imputation sequence, were sex (male/female), marital status (married, divorced/separated/widowed, or single), ethnicity (white/non-white), age in years (minimum 18 years, maximum 110 years), response to question on perceived need to change weight, height (minimum 122 cm, maximum 214 cm), weight (minimum 40 kg, maximum 200 kg), highest level of education (4 levels), cancer spread (yes/no), and four variables for treatment type (surgery, chemotherapy, radiotherapy, and hormone therapy). Missing data analysis found that 2.96% of 75,855 values for these variables were missing, and 27.8% cases had at least one missing value. Little’s *t* test demonstrated that the data were not missing completely at random. The multiple imputation was run twice and the analysis run on both datasets. The results were similar and can therefore be considered to have converged so it was concluded that five imputations was adequate. The results from the first imputation are presented. After multiple imputation, the pooled data indicated that only 80 participants (1.4%) had a BMI in the underweight category, therefore this group was excluded from further analysis to reduce risk of bias. A sensitivity analysis was performed to compare the regression model for each cancer type with the findings for the total sample. The results for each cancer type were found to be consistent with the overall findings (Supplementary material: Table 4).

Significance was assessed at the 0.05 level for all statistical tests.

## Results

### Sample characteristics

A total of 5835 questionnaires were returned from the 13,500 questionnaires that were sent out (43% response rate). Participant characteristics are presented in Table 1. The mean age was 67.4 years, 56% were female, and 90% were white. The mean BMI was 27, and 59.2% were classified as overweight or obese. The three most commonly reported comorbidities among participants in this study were arthritis (*N* = 1535, 26.3%), diabetes (*N* = 717, 12.3%), and another cancer (*N* = 709, 12.2%).

**Table 1:** Demographic and clinical characteristics of participants living with breast, prostate, and colorectal cancer

		Total *N* = 5835	Healthy weight *N* = 1978	Overweight *N* = 2247	Obese *N* = 1209
Age in years (mean, SD)		67.43 (11.818)	67.4 (13.089)	67.86 (10.87)	66.16 (10.678)
Missing data (*N*, %)		36 (0.6)	12 (0.6)	10 (0.4)	5 (0.4)
Gender (*N*, %)					
Male		2553 (43.8)	751 (38)	1157 (51.5)	510 (42.2)
Female		3266 (56)	1221 (61.7)	1083 (48.2)	698 (57.7)
Missing data		16 (0.3)	6 (0.3)	7 (0.3)	1 (0.1)
Ethnicity (*N*, %)					
White		5249 (90)	1799 (91)	2038 (90.7)	1098 (90.8)
Non-white		554 (9.5)	171 (8.6)	201 (8.9)	108 (8.9)
Missing data		32 (0.5)	8 (0.4)	8 (0.4)	3 (0.2)
Highest level of education (*N*, %)					
No formal qualifications		1709 (29.3)	492 (24.9)	670 (29.8)	408 (33.7)
GCSE/vocational		1613 (27.6)	521 (26.3)	631 (28.1)	366 (30/3)
A-level		584 (10)	220 (11.1)	211 (9.4)	121 (110)
Degree or higher		1379 (23.6)	562 (28.4)	504 (22.4)	220 (18.2)
Missing data		550 (9.4)	183 (9.3)	231 (10.3)	94 (7.8)
Marital status (*N*, %)					
Married/cohabiting		4037 (69.2)	1358 (68.7)	1616 (71.9)	831 (68.7)
Separated/divorced/widowed/single		1781 (30.5)	614 (31)	627 (27.9)	376 (31.1)
Missing data		17 (0.3)	6 (0.3)	4 (0.2)	2 (0.2)
Employment situation (*N*, %)					
Employed		1684 (28.9)	604 (30.5)	636 (28.3)	345 (28.5)
Retired		3595 (61.6)	1212 (61.3)	1426 (63.5)	717 (59.3)
Other		502 (8.6)	144 (7.3)	167 (7.4)	138 (11.4)
Missing data		54 (0.9)	18 (0.9)	18 (0.8)	9 (0.7)
Cancer type (*N*, %)					
Breast		2786 (47.7)	1024 (51.8)	943 (42)	593 (49)
Prostate		1839 (31.5)	530 (26.8)	840 (37.4)	366 (30.3)
Colorectal		1210 (20.7)	424 (21.4)	464 (20.6)	250 (20.7)
Missing		0	0	0	0
BMI (mean, SD)		27 (4.9)	–	–	–
Missing data (*N*, %)		336 (5.8)	–	–	–
BMI category (*N*, %)					
Underweight		65 (1.1)	–	–	–
Healthy weight		1978 (33.9)	–	–	–
Overweight		2247 (38.5)	–	–	–
Obese		1209 (20.7)	–	–	–
Missing data		336 (5.8)	–	–	–
Cancer spread (*N*, %)					
Yes		558 (9.6)	187 (9.5)	224 (10)	118 (9.8)
No		4498 (77.1)	1548 (78.3)	1727 (76.9)	920 (76.1)
Don’t know		373 (6.4)	114 (5.8)	145 (6.5)	85 (7.0)
Missing data		406 (7)	129 (6.5)	151 (6.7)	86 (7.1)
Comorbidity (*N*, %)					
Osteoporosis		530 (9.1)	219 (11.1)	171 (7.6)	88 (7.3)
Diabetes		717 (12.3)	140 (7.1)	266 (11.8)	246 (20.3)
Asthma		589 (10.1)	169 (8.5)	212 (9.4)	156 (12.9)
Emotional or psychiatric illness		404 (6.9)	123 (6.2)	145 (6.5)	112 (9.3)
Stroke		181 (3.1)	52 (2.6)	67 (3)	46 (3.8)
Parkinson’s disease		36 (0.6)	10 (0.5)	15 (0.7)	7 (0.6)
Alzheimer’s/dementia		31 (0.5)	8 (0.4)	14 (0.6)	5 (0.4)
Lung disease		248 (4.3)	81 (4.1)	85 (3.8)	54 (4.5)
Arthritis		1535 (26.3)	413 (20.9)	5546 (24.3)	448 (37.1)
Angina		208 (3.6)	67 (3.4)	86 (3.8)	45 (3.7)
Heart attack		240 (4.1)	84 (4.2)	92 (4.1)	55 (4.5)
Heart murmur		158 (2.7)	57 (2.9	56 (2.5)	39 (3.2)
Irregular heart rhythm		522 (8.9)	180 (9.1)	191 (8.5)	114 (9.4)
Any other heart trouble		211 (3.6)	68 (3.4)	86 (3.8)	45 (3.7)
Another cancer		709 (12.2)	227 (11.5)	270 (12)	160 (13.2)
Hypertension		159 (2.7)	34 (1.7)	66 (2.9)	52 (4.3)
Number of comorbidities (*N*, %)					
0		1849 (31.7)	718 (36.3)	740 (32.9)	282 (23.3)
1		1991 (34.1)	685 (34.6)	778 (34.6)	395 (32.7)
2		1120 (19.2)	336 (17.0)	423 (18.8)	279 (23.1)
3 +		875 (15)	239 (12.1)	306 (13.6)	253 (20.9)
Treatment					
Surgery		4065 (69.7)	1424 (73)	1531 (68.9)	839 (70.1)
Chemotherapy		1824 (31.3)	622 (32)	669 (30.2)	404 (33.8)
Radiotherapy		3348 (57.4)	1101 (56.5)	1285 (57.7)	747 (62.4)
Hormone therapy		1895 (32.5)	659 (33.9)	723 (32.6)	400 (33.4)

Abbreviations: *BMI*, body mass index; *GCSE*, General Certificate of Secondary Education

### Chi-squared test analyses

There were significant differences in perceptions of a need to change weight between participants in healthy weight, overweight, and obese BMI categories. Table 2 shows that participants in each BMI category differed significantly from each other in thinking they should lose, gain, or not change their weight (*P* < 0.001). There was no difference in the proportion of participants in each BMI category who did not know whether they should be trying to change their weight.

**Table 2:** Frequency distribution of responses of participants in the healthy, overweight, and obese BMI categories on whether they thought they should change their weight, with chi-square tests (*N* = 5756)

	Weight classification according to BMI			
Perceived need to change weight	Healthy weight (*N* = 2078) Count (%)	Overweight (*N* = 2374) Count (%)	Obese (*N* = 1304) Count (%)	*P*
Lose weight (*N* = 3076)	462 (22.2)	1499 (63.1)	1116 (85.6)	< 0.001*
Gain weight (*N* = 151)	124 (6)	21 (0.9)	7 (0.5)	< 0.001*
Not change (*N* = 2284)	1416 (68.1)	739 (31.1)	127 (9.7)	< 0.001*
Don’t know (*N* = 244)	75 (3.6)	115 (4.8)	54 (4.1)	0.111

Numbers are rounded from pooled multiply imputed data, hence cells may not sum to column and row totals

*Significant at the 0.05 level

Shaded cells indicate participants whose perception of whether they should change their weight was in line with WCRF recommendations to achieve a BMI of ≥ 18.5 and < 25

Abbreviations: *BMI*, body mass index

For the majority of participants, perceived need for weight change was in line with WCRF recommendations to achieve a BMI of ≥ 18.5 and < 25 (shaded cells in Table 2), though these proportions differed between healthy weight (68%), overweight (63%) and obese (86%) categories. Ten percent of participants in the obese group and 31% in the overweight group did not perceive a need for weight loss. Repeating this analysis with a dataset only including participants with complete data for height, weight, and perception of a need to change weight produced similar distributions and chi-square test results (Supplementary Material: Table 5).

### Logistic regression analysis

Among participants in overweight and obese categories, those who were older, male, non-white, not married or cohabiting, had no formal qualifications, whose cancer had spread, and who had received chemotherapy had greater odds of not perceiving a need to lose weight (Table 3). Male participants were 56% more likely to not perceive a need to lose weight than female participants (95% CI, 1.32–1.84). Non-white participants were 66% more likely to not perceive a need to lose weight than white participants (95% CI, 1.27–2.17). Non-married or cohabiting participants were 31% more likely not perceive a need to lose weight than married and cohabiting participants (95% CI, 1.11–1.56). Participants with no formal qualifications, were 52% more likely to not perceive a need to lose weight than those with a degree or above (95% CI, 1.19–1.93). Participants whose cancer had spread were 50% more likely to not perceive a need to lose weight than those whose cancer had not spread (95% CI, 1.16–1.93). Participants with 3 or more comorbidities had lower odds of not perceiving a need to lose weight, therefore individuals with additional comorbidities had increased odds of perceiving a need for weight loss. Participants who had received chemotherapy were 24% more likely to not perceive a need for weight loss than those who had not received chemotherapy (95% CI 1.01–1.53), whereas participants who had received radiotherapy or hormone therapy were 19% and 18% more likely to perceive a need to lose weight than participants who had received these treatments (95% CI 0.69–0.96 and 0.68–0.99 respectively for not perceiving a need to lose weight). Repeating this analysis with a second imputed dataset, and then with the dataset only including completers (Supplementary Material: Table 6) showed the same associations.

**Table 3:** Logistic regression model for not perceiving a need to lose weight in participants with overweight and obesity (*N* = 1065)

Variables		OR	CI	*P*
Age (continuous, in years)		1.07	1.06–1.08	< 0.001*
Gender				
Female		1.00	–	–
Male		1.62	1.34–1.96	< 0.001*
Ethnicity				
White		1.00	–	–
Any other ethnicity		1.68	1.25–2.25	0.001*
Marital status				
Married/cohabiting		1.00	–	–
Separated/divorced/widowed/single		1.32	1.11–1.57	0.002*
Highest education				
Degree or above		1.00	–	–
A-Level		1.06	0.76–1.49	0.717
GCSE/vocational		1.22	0.95–1.57	0.115
No formal qualifications		1.51	1.20–1.91	0.001*
Cancer spread				
No		1.00	–	–
Yes		1.50	1.10–2.04	0.011*
Comorbidities				
0		1.00	–	-
1		0.93	0.76–1.15	0.523
2		0.80	0.63–1.01	0.058
3 +		0.76	0.59–0.97	0.029*
Treatment				
Surgery		1.13	0.93–1.38	0.222
Radiotherapy		0.81	0.69–0.96	0.015*
Chemotherapy		1.24	1.01–1.53	0.043*
Hormone therapy		0.82	0.68–0.99	0.036*

*Significant at the 0.05 level

Abbreviations: *BMI*, body mass index; *GCSE*, General Certificate of Secondary Education

### Discussion

The majority of participants with obesity (85%), and overweight (64%) perceived a need to lose weight. Among those in the overweight or obese categories, not perceiving a need to lose weight was associated with being older, male, non-white, not being married or cohabiting, having no formal qualifications (compared to having a degree), having cancer that had spread, having no comorbidities (compared to 3 or more), and having had chemotherapy.

Prevalence of obesity in this sample was 20.7%, lower than the 31.7% previously reported in cancer survivors in the United States [29]. Prevalence of obesity in this sample was also lower than in the general adult population in the United Kingdom, which is estimated to be 28% [30]. In this study, the greatest proportion of participants who thought they should lose weight were in the obese category, followed by the overweight, then healthy weight categories, which is in line with other studies exploring associations between BMI and desire to lose weight [31, 32].

Eighty-five percent of participants in the obese category thought they should be trying to lose weight. This is lower than levels of desire to lose weight among adults with obesity in the general population (94.2%) [33], but considerably higher than levels of accuracy in self-perception of obesity in other studies [19, 34], though it is important to make the distinction between perceiving oneself as having obesity, and perceiving a need to lose weight. For example, participants in this study who thought they should be trying to lose weight may not have perceived themselves as having overweight or obesity. The proportion of participants in the overweight category who thought they should be trying to lose weight (63%) was also lower than the proportion of those with overweight in the general population reporting a desire to lose weight (79.4%) [33]. The overweight category had the lowest proportion of participants whose perceptions of whether they should change their weight were in line with WCRF recommendations of having a BMI within the healthy range, and over 30% participants in this category reported that they did not think they needed to change their weight. A potential explanation for this is that individuals in the overweight category may not have discussed weight with healthcare professionals. Alternatively, they may have been advised not to pursue weight loss by healthcare professionals, friends, and/or family. It is also possible that a subset of participants in overweight and obese categories do perceive themselves as being in these BMI categories, but still do not perceive a need to lose weight due to health or cultural reasons.

Nevertheless, the vast majority of participants with overweight and obesity in this sample did perceive a need to lose weight, suggesting these individuals may be receptive to interventions seeking to support weight management. However, there is currently a lack of programs designed specifically to support people LWBC. The lack of available support to achieve weight loss may be detrimental to the wellbeing of this group and could contribute to negative feelings and frustration if they are unsuccessful in achieving weight loss despite perceiving a need for it.

Nearly a quarter of healthy weight participants thought they should be trying to lose weight. Though the desire for people LWBC of a healthy weight to lose weight could be seen as concerning, this figure is lower than the reported 35.3% of healthy weight individuals in the general population who want to lose weight [33]. The lower proportion among this patient population may be due to medical advice against unnecessary weight loss, as well as the high average age of participants, as concern about weight is known to decrease with increasing age [35]. However, more research to understand the rationale behind perceiving a need to lose weight in people LWBC of a healthy weight is needed to prevent unnecessary and potentially detrimental weight loss. Furthermore, a future study comparing perceptions of weight, perceived need to change weight, and proportions of individuals who are actively trying to change or maintain their weight, including with analysis across BMI categories, may identify groups who need support actioning this change, as well as those trying to change their weight in a way that may be detrimental to their health.

The regression analysis highlighted demographic and health factors associated with not perceiving a need to lose weight in participants with overweight and obesity. These were older age, being male, non-white, and not married or cohabiting, having no formal qualifications (compared to having a degree), having cancer that had spread, having no comorbidities (compared to having 3 or more comorbidities), and having received chemotherapy. Previous studies have identified associations between older age [25, 36], being male [25], being non-white [25], and having lower levels of education [36] in misperception, particularly underestimation, of weight status, but few studies have investigated perceived need to change weight. These factors may describe a subset of the population who are at risk of health inequalities, which may be prevented through increased awareness of own weight status, and risks associated with overweight and obesity, as well as tailored support in achieving weight loss. Research to understand the reasons for not perceiving a need to lose weight among these groups is needed, as is research on effective targeted support and advice for those with overweight and obesity who underestimate their weight status, and/or who do not perceive a need to lose weight.

The finding that individuals with comorbidities are more likely to perceive a need to lose weight is at odds with previous findings that weight misperception was more common in those with a diagnosed chronic condition [36], though it is again important to make the distinction between weight perception and perceived need to lose weight. Neither the present nor afore mentioned studies collected data on the date of development of comorbid diseases in relation to weight status, therefore interpreting these findings is problematic. With this in mind, some possible explanations for the findings of this study may be carefully considered. It is possible that the care pathways of conditions other than cancer are more successful in raising awareness of weight status in patients and advising them on actions to reduce risks. This is supported by evidence that awareness of having hypertension and dyslipidaemia were negatively associated with weight underestimation [34], suggesting people may be more aware of the associations of overweight and obesity with these conditions than with cancer. The two most commonly reported comorbidities among participants in the present study were arthritis and diabetes, both of which are associated with overweight and obesity [37, 38]; therefore, healthcare professionals may have advised participants to lose weight to improve their symptoms. However, there is little evidence that diagnosis of comorbid diseases is a successful motivator for weight loss. Future studies using qualitative methods to understand the role of comorbidities in perception of weight status and need to lose weight among people LWBC may improve our understanding of this association.

This study also found differences in perceived need to lose weight among individuals with overweight or obesity who had undergone different cancer treatments. Those who had undergone chemotherapy were significantly less likely to perceive a need to lose weight than those who had not, and those who had received radiotherapy or hormone therapy were significantly more likely to perceive a need to lose weight than those who had not. The reason for these differences in unclear, as to date there has been limited research comparing how treatment types may differentially impact individuals’ perception of a need to lose weight, and factors which may impact this. The survey used in this study asked participants to self-report the treatment types they had received using broad check boxes (e.g. surgery/chemotherapy), without providing further detail. Therefore, any inferences about how specific treatment type affects perceived need to lose weight must be made with caution. Future studies focusing on differential effects of different treatment types could explore these associations further.

When considering the clinical and policy implications of these findings we must consider how beneficial it is to increase perceptions of a need to lose weight among individuals LWBC with overweight and obesity. Some studies have found positive associations between weight loss and mortality among breast cancer survivors with overweight and obesity [6, 39], but interpreting these findings is challenging as few studies make the distinction between intentional and disease-related (unintentional) weight loss. A large study using data from three longitudinal cohorts also found that those who perceived themselves as having overweight were at an increased risk of subsequent weight gain, and that this effect was predicted by stress-induced eating [40]. However, those participants were not cancer survivors, who may have different motivations for achieving a healthy BMI. Nevertheless, these studies highlight potential consequences of increasing the proportion of individuals with overweight and obesity who perceive a need for weight loss, especially those who may not need or benefit from weight loss, and in the absence of weight loss interventions tailored for people LWBC. As a recent Cochrane review found that tailored weight loss interventions for people LWBC can lead to significant improvements in anthropometric measures and aspects of quality of life, it seems that there is benefit to participation in such interventions, and further development of weight loss interventions for people LWBC using evidence-based techniques based on behavior change theory is necessary to appropriately support this group.

This study has a number of strengths and limitations. One strength is the large sample size of 5835 participants. The study also collected data on perceived need to lose weight, which has been minimally explored to date among people LWBC. Limitations included self-reporting of height and weight data to calculate BMI, which may be associated with under-reporting of weight, particularly in individuals with overweight and obesity [41]. BMI is also an imperfect indicator of nutritional status, especially in individuals LWBC for whom cancer cachexia is a concern [42]. Non-white ethnicities were also underrepresented in the sample. Additionally, participants who ignored or skipped the question on comorbidities were coded as having 0 comorbidities, which may be inaccurate. Furthermore, the survey used in this study did not collect detail on cancer treatment beyond asking participants to select from a list of treatments which they had received, limiting inferences which could be made on the potential effects of treatment type of perception on a need to lose weight.

In conclusion, individuals LWBC with obesity are the most likely to perceive a need to lose weight, followed by those in the overweight, then healthy weight BMI categories. Not perceiving a need to change weight in those with overweight is common, and more research is needed to understand the rationale for this in this population. Among those with overweight and obesity, factors associated with not perceiving a need to lose weight included demographic and health variables, which may indicate a subset of people LWBC who need additional advice and support with weight management. Development and increased availability of weight management programs for people LWBC is needed.

### Supplementary Information

Below is the link to the electronic supplementary material.

Supplementary file1 (DOCX 54 KB)

## Data Availability

The datasets generated during and/or analyzed during the current study are available from the corresponding author on reasonable request.
