# Supplementary material for: Perception of a need to change weight in individuals living with and beyond breast, prostate and colorectal cancer: a cross-sectional survey
Source: J Cancer Surviv. 2023 Jan 26;18(3):844–53. doi: 10.1007/s11764-023-01333-0 (PMC11081928; doi:10.1007/s11764-023-01333-0)
Supplement: Supplementary file 1 — Supplementary file1 (DOCX 54 KB) [file 11764_2023_1333_MOESM1_ESM.docx]

# Supplementary material

Table 4 Sensitivity analysis for regression analysis for not perceiving a need to lose weight in participants with overweight and obesity with breast (N=342), prostate (N=444), and colorectal cancer (N=277)

| **Variables** | | **OR** | | | | **CI** | | | | **P** | | | |
| --- | --- | --- | --- | --- | --- | --- | --- | --- | --- | --- | --- | --- | --- |
|  | | Full | Breast | Prostate | Colorectal | Full | Breast | Prostate | Colorectal | Full | Breast | Prostate | Colorectal |
| **Age** (continuous, in years) | | 1.07 | 1.09 | 1.06 | 1.05 | 1.06-1.08 | 1.07-1.11 | 1.06-1.08 | 1.03-1.07 | <0.001* | <0.001* | <0.001* | <0.001* |
| **Gender** | |  |  |  |  |  |  |  |  |  |  |  |  |
|  | Female | 1.00 |  |  | 1.00 | - |  |  | - | - |  |  | - |
|  | Male | 1.62 |  |  | 1.98 | 1.34-1.96 |  |  | 1.39-2.82 | <0.001* |  |  | <0.001* |
| **Ethnicity** | |  |  |  |  |  |  |  |  |  |  |  |  |
|  | White | 1.00 | 1.00 | 1.00 | 1.00 | - | - | - | - | - | - | - | - |
|  | Any other ethnicity | 1.68 | 1.98 | 1.53 | 1.48 | 1.25-2.25 | 1.25-3.14 | 0.99-2.36 | 0.59-3.70 | 0.001* | 0.004* | 0.053 | 0.394 |
| **Marital status** | |  |  |  |  |  |  |  |  |  |  |  |  |
|  | Married/cohabiting | 1.00 | 1.00 | 1.00 | 1.00 | - | - | - | - | - | - | - | - |
|  | Separated/divorced/widowed/single | 1.32 | 1.61 | 1.21 | 0.96 | 1.11-1.57 | 1.22-2.11 | 0.91-1.62 | 0.66-1.40 | 0.002* | 0.001* | 0.190 | 0.846 |
| **Highest education** | |  |  |  |  |  |  |  |  |  |  |  |  |
|  | Degree or above | 1.00 | 1.00 | 1.00 | 1.00 | - | - | - | - | - | - | - | - |
|  | A-Level | 1.06 | 1.72 | 0.80 | 0.79 | 0.76-1.49 | 0.96-3.12 | 0.48-1.33 | 0.38-1.64 | 0.717 | 0.070 | 0.390 | 0.520 |
|  | GCSE/vocational | 1.22 | 1.58 | 1.13 | 0.91 | 0.95-1.57 | 1.07-2.34 | 0.72-1.77 | 0.55-1.49 | 0.115 | 0.022* | 0.598 | 0.703 |
|  | No formal qualifications | 1.51 | 1.89 | 1.32 | 1.39 | 1.20-1.91 | 1.25-2.86 | 0.91-1.90 | 0.87-2.24 | 0.001* | 0.003* | 0.138 | 0.169 |
| **Cancer spread** | |  |  |  |  |  |  |  |  |  |  |  |  |
|  | No | 1.00 | 1.00 | 1.00 | 1.00 | - | - | - | - | - | - | - | - |
|  | Yes | 1.50 | 2.00 | 1.04 | 1.62 | 1.10-2.04 | 1.18-3.40 | 0.65-1.67 | 1.97-2.71 | 0.011* | 0.011* | 0.876 | 0.066 |
| **Comorbidities** | |  |  |  |  |  |  |  |  |  |  |  |  |
|  | 0 | 1.00 | 1.00 | 1.00 | 1.00 | - | - | - | - | - | - | - | - |
|  | 1 | 0.93 | 1.05 | 0.88 | 0.90 | 0.76-1.15 | 0.72-1.53 | 0.65-1.20 | 0.60-1.37 | 0.523 | 0.802 | 0.427 | 0.635 |
|  | 2 | 0.80 | 0.89 | 0.73 | 0.76 | 0.63-1.01 | 0.59-1.35 | 0.51-1.05 | 0.46-1.26 | 0.058 | 0.592 | 0.089 | 0.286 |
|  | 3+ | 0.76 | 0.63 | 0.93 | 0.67 | 0.59-0.97 | 0.41-0.99 | 0.63-1.38 | 0.40-1.11 | 0.029* | 0.044* | 0.733 | 0.122 |
| **Treatment** | |  |  |  |  |  |  |  |  |  |  |  |  |
|  | Surgery | 1.13 | 0.94 | 1.10 | 1.08 | 0.93-1.38 | 0.61-1.45 | 0.81-1.49 | 0.65-1.79 | 0.222 | 0.786 | 0.536 | 0.767 |
|  | Radiotherapy | 0.81 | 1.00 | 0.73 | 0.77 | 0.69-0.96 | 0.71-1.41 | 0.56-0.95 | 0.50-1.21 | 0.015* | 0.985 | 0.019* | 0.259 |
|  | Chemotherapy | 1.24 | 1.20 | 1.86 | 1.05 | 1.01-1.53 | 0.86-1.69 | 0.94-3.69 | 0.72-1.53 | 0.043* | 0.286 | 0.073 | 0.812 |
|  | Hormone therapy | 0.82 | 0.88 | 0.83 | 1.29 | 0.68-0.99 | 0.66-1.18 | 0.62-1.12 | 0.49-3.35 | 0.036* | 0.393 | 0.226 | 0.604 |

*Significant at the 0.05 level

Table 5: Frequency distribution of responses of participants in the healthy, overweight and obese BMI categories on whether they thought they should change their weight, with chi-square tests for participants with complete data for BMI and perceived need to change weight (N=5,394).

|  | Weight classification according to BMI | | |  |
| --- | --- | --- | --- | --- |
| Perceived need to change weight | Healthy weight (N=1,969)  Count (%) | Overweight (N=2,225)  Count (%) | Obese (N=1,200)  Count (%) | P |
| Lose weight (N=2,891) | 440 (22.3) | 1420 (63.8) | 1031 (85.9) | <0.001* |
| Gain weight (N=144) | 118 (6) | 19 (0.9) | 7 (0.6) | <0.001* |
| Not change (N=2,154) | 1349 (68.5) | 689 (31) | 116 (9.7) | <0.001* |
| Don’t know (N=205) | 62 (3.1) | 97 (4.4) | 46 (3.8) | 0.123 |

*Significant at the 0.05 level

Shaded cells indicate participants whose perception of whether they should change their weight was in line with WCRF recommendations to achieve a BMI of ≥18.5 and <25.

Abbreviations: BMI, body mass index

Table 6: Logistic regression model for not perceiving a need to lose weight in participants with overweight and obesity with complete data for predictor variables in model (N=706).

| **Variables** | | **OR** | **CI** | **P** |
| --- | --- | --- | --- | --- |
| **Age** (continuous, in years) | | 1.07 | 1.06-1.08 | <0.001* |
| **Gender** | |  |  |  |
|  | Female | 1.00 | - | - |
|  | Male | 1.73 | 1.37-2.17 | <0.001* |
| **Ethnicity** | |  |  |  |
|  | White | 1.00 | - | - |
|  | Any other ethnicity | 1.70 | 1.22-2.36 | 0.002* |
| **Marital status** | |  |  |  |
|  | Married/cohabiting | 1.00 | - | - |
|  | Separated/divorced/widowed/single | 1.38 | 1.12-1.69 | 0.003* |
| **Highest education** | |  |  |  |
|  | Degree or above | 1.00 | - | - |
|  | A-Level | 1.03 | 0.71-1.50 | 0.860 |
|  | GCSE/vocational | 1.22 | 0.93-1.60 | 0.144 |
|  | None | 1.50 | 1.16-1.96 | 0.002* |
| **Cancer spread** | |  |  |  |
|  | No | 1.00 | - | - |
|  | Yes | 1.60 | 1.17-2.19 | <0.003* |
| **Comorbidities** | |  |  |  |
|  | 0 | 1.00 | - | - |
|  | 1 | 0.89 | 0.70-1.12 | 0.323 |
|  | 2 | 0.74 | 0.56-0.97 | 0.031* |
|  | 3+ | 0.71 | 0.53-0.96 | 0.025* |
| **Treatment** | |  |  |  |
|  | Surgery | 1.03 | 0.81-1.32 | 0.785 |
|  | Radiotherapy | 0.81 | 0.66-0.99 | 0.038* |
|  | Chemotherapy | 1.30 | 1.03-1.66 | 0.030* |
|  | Hormone therapy | 0.83 | 0.66-1.04 | 0.106 |
|  |  |  |  |  |

*Significant at the 0.05 level

Abbreviations: BMI, body mass index; GCSE, General Certificate of Secondary Education.
